# Non-monotonic temperature dependence of radiation defect dynamics in silicon carbide

**DOI:** 10.1038/srep30931

**Published:** 2016-08-03

**Authors:** L. B. Bayu Aji, J. B. Wallace, L. Shao, S. O. Kucheyev

**Affiliations:** 1 Lawrence Livermore National Laboratory, Livermore, California 94550, USA; 2 Department of Nuclear Engineering, Texas A&M University, College Station, Texas 77843, USA

## Abstract

Understanding response of solids to particle irradiation remains a major materials physics challenge. This applies even to SiC, which is a prototypical nuclear ceramic and wide-band-gap semiconductor material. The lack of predictability is largely related to the complex, dynamic nature of radiation defect formation. Here, we use a novel pulsed-ion-beam method to study dynamic annealing in 4*H*-SiC ion-bombarded in the temperature range of 25–250 °C. We find that, while the defect recombination efficiency shows an expected monotonic increase with increasing temperature, the defect lifetime exhibits a non-monotonic temperature dependence with a maximum at ~100 °C. This finding indicates a change in the dominant defect interaction mechanism at ~100 °C. The understanding of radiation defect dynamics may suggest new paths to designing radiation-resistant materials.

Silicon carbide (SiC) is an important material for electronics^1^,^2^ and nuclear materials^3^,^4^ technologies. For both of these applications, the fundamental understanding of radiation damage processes is highly desirable, particularly for practically-relevant irradiation at room temperature (*T*) and above. For such irradiation conditions, all three main commonly available polymorphs of SiC (3*C*, 4*H*, and 6*H*) exhibit pronounced dynamic annealing (DA)^5^,^6^,^7^,^8^,^9^,^10^,^11^,^12^,^13^,^14^,^15^, which refers to migration, recombination, and clustering of radiation-generated point defects *during* irradiation. The degree of DA increases with increasing sample *T*. This is evidenced as a reduction in the level of stable post-irradiation disorder or as an increase in the ion dose required to reach a certain level of disorder with increasing *T*^5^,^6^,^7^,^8^,^10^,^12^,^15^. The DA also manifests as a dose rate dependence of damage (with all the other irradiation conditions being constant)^11^,^13^,^14^,^15^. However, due to complexity of radiation defect interaction processes and the lack of direct experimental methods to study them, mechanisms of DA in SiC remain elusive. Indeed, physically very different models of point defect interaction can describe the same experimental *T*-dependencies of the amorphization dose^16^. More sophisticated measurements of radiation defect dynamics are needed to unravel the underlying mechanisms.

We have recently demonstrated^11^,^17^,^18^,^19^ that details of DA can be accessed in pulsed beam experiments when the total ion dose is delivered as a train of equal square pulses with a duration of *t*_*on*_ and an instantaneous dose rate of *F*_*on*_ separated by a passive portion of the beam duty cycle of *t*_*off*_ (see the inset in Fig. 1). In particular, the dependence of the amount of stable post-irradiation disorder on *t*_*off*_ reveals the characteristic time constant of DA (*τ*) and, hence, the DA rate^17^. We have successfully applied such a pulsed-beam method to measure *τ* in Si at room *T* and in 3*C*-SiC at 100 °C^11^,^17^,^19^. Here, we report the *τ*(*T*) dependence in 4*H*-SiC bombarded with energetic Ar ions in the *T* range of 25–250 °C. Our results reveal a non-monotonic *τ*(*T*) dependence with a maximum of ~5 ms at ~100 °C, reflecting a change in the dominant DA mechanism at ~100 °C. This result has important practical implications since design strategies for radiation-tolerant materials are determined by the dominant defect formation and DA processes.

## Results and Discussion

**Figure 1:**
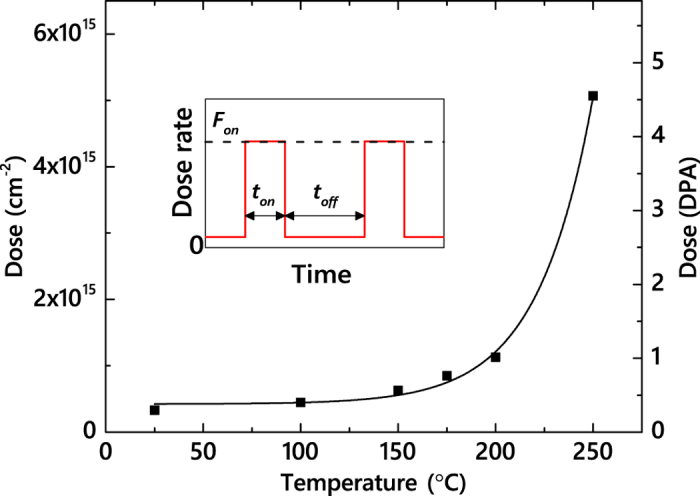
Temperature dependence of the ion dose required to reach a damage level of 0.7 in 4*H*-SiC bombarded with a continuous beam (i.e., *t*_*off*_ = 0) of 500 keV Ar ions with a dose rate of 1.7 × 10^13^ cm^−2^ s^−1^. The solid line is an exponential fit to the data. The inset is a schematic of the time dependence of the instantaneous dose rate for pulsed beam irradiation, defining *t*_*on*_, *t*_*off*_, and *F*_*on*_.

Figure 1 shows an exponential *T* dependence of the dose required to achieve an averaged bulk disorder level (*n*) of 0.7 (with *n* = 1 corresponding to complete amorphization) for continuous beam irradiation (i.e., *t*_*off*_ = 0) of 4*H*-SiC. Such a dose increases from 0.2 to 4.3 displacements per atom (DPA) when *T* is increased from 25 to 250 °C, reflecting the thermally activated nature of DA processes. An increase in the dose required to reach a certain disorder level with increasing *T* is expected^5^,^6^,^7^,^8^,^9^,^10^,^12^,^15^. Indeed, at higher *T*s, point defects have higher diffusivity and experience more efficient vacancy-interstitial recombination. We, however, note that the doses revealed by Fig. 1 are smaller than those reported in the previous studies of SiC (refs 5, 6, 7, 8, 9, 10,15) due to a larger dose rate used in the present work. As we discussed in detail in our recent systematic study of the damage buildup in 3*C*-SiC^12^, this highlights the dominant role of DA in the formation of stable post-irradiation damage in SiC at elevated *T*s. Based on damage buildup data such as shown in Fig. 1, we have chosen ion doses for pulsed beam experiments at different *T*s so that, for *t*_*off*_ = 0 (i.e., continuous beam irradiation), *n* is in the range of 0.6–0.8, which is a nonlinear regime of damage buildup with pronounced DA^11^,^12^,^20^.

**Figure 2:**
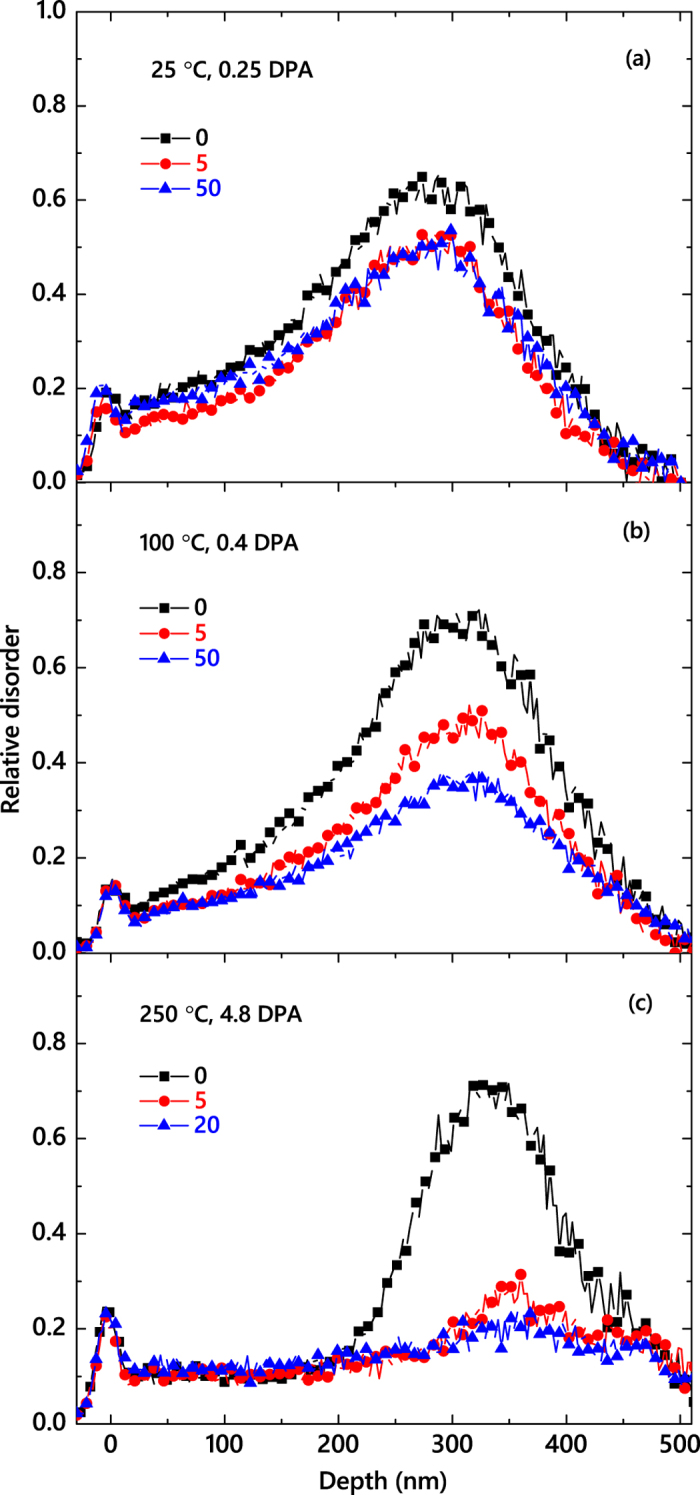
Selected depth profiles of relative disorder in 4*H*-SiC bombarded with a pulsed beam of 500 keV Ar ions with *F*_*on*_ = 1.7 × 10^13^ cm^−2^ s^−1^, *t*_*on*_ = 1 ms, and different *t*_*off*_ values, given in legends (in units of ms), at different temperatures and total doses, also shown in the legends. For clarity, only every 5th experimental point is depicted.

Figure 2 shows representative depth profiles of relative disorder for bombardment of 4*H*-SiC with continuous or pulsed beams at *T*s of 25, 100, and 250 °C. Each panel of Fig. 2 shows data for cases when all the irradiation conditions are kept constant except for *t*_*off*_. It is seen that, for all three *T*s shown, depth profiles are bimodal, with the first small peak at the sample surface and the second major peak in the crystal bulk. The bulk peak (with a height of *n*) is centered on ~300 nm, which corresponds to the maximum of the nuclear energy loss profile for 500 keV Ar ions. It is also seen from Fig. 2 that *n* decreases with increasing *t*_*off*_ for all three *T*s. We have found such a reduction in *n* with increasing *t*_*off*_ in all our measurements at different *T*s. Figure 2 further shows that the degree of reduction in *n* with increasing *t*_*off*_ increases dramatically with increasing *T*. For example, for *T* = 250 °C [Fig. 2(c)], an increase in *t*_*off*_ from 0 to 5 ms results in an ~2.5-fold reduction in *n*. In contrast, the damage level at the sample surface is independent of *t*_*off*_, suggesting different mechanisms of bulk and surface disordering^11^,^17^,^19^,^21^.

**Figure 3:**
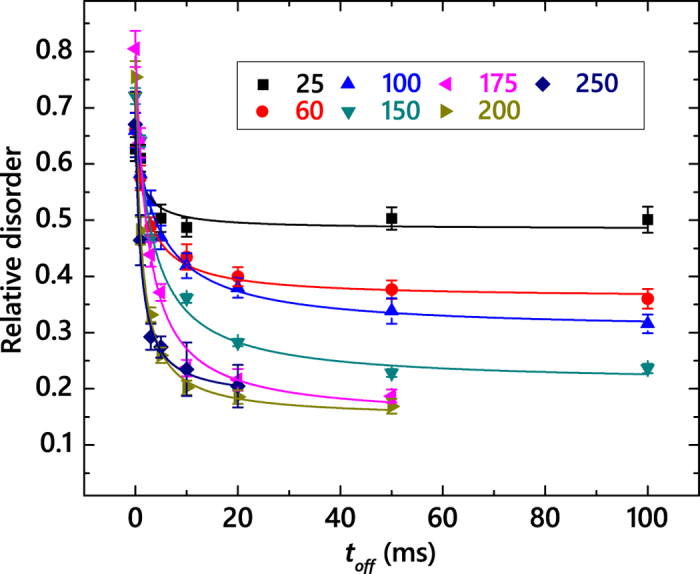
Level of relative bulk disorder in 4*H*-SiC bombarded with 500 keV Ar ions with *F*_*on*_ = 1.7 × 10^13^ cm^−2^ s^−1^ and *t*_*on*_ = 1 ms as a function of the passive portion of the beam duty cycle (*t*_*off*_) at different temperatures, given in the legend (in units of °C). Fitting curves of the data with the second order decay equation are shown by solid lines.

Figure 3 summarizes *n*(*t*_*off*_) dependencies for all the *T*s of this study (25–250 °C). It reveals that *n* monotonically decreases with increasing *t*_*off*_ in all these cases. Solid lines in Fig. 3 are fits of the data via the Marquardt-Levenberg algorithm with the second order decay equation 
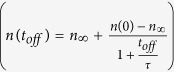
. Here, *n*_∞_ is relative disorder for *t*_*off*_  ≫ *τ*. We have found that all the *n*(*t*_*off*_) dependencies from Fig. 3 obey the second order decay better than the first order (i.e., exponential) decay.

**Figure 4:**
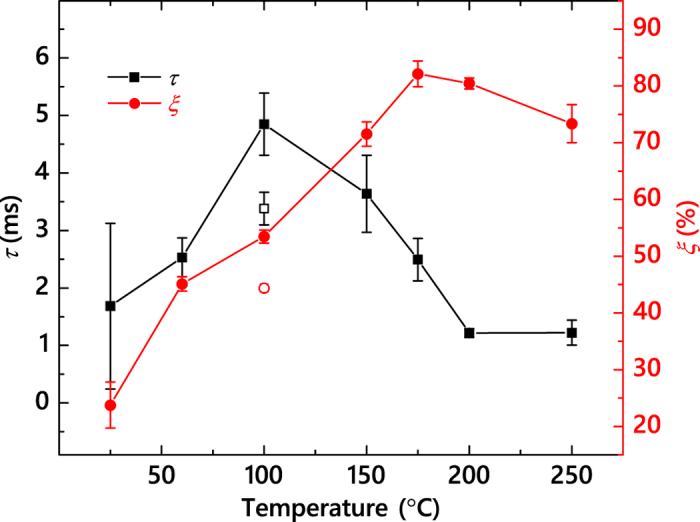
Closed symbols: temperature dependencies of the effective time constant of DA (*τ*) and the DA efficiency (*ξ*) for 4*H*-SiC bombarded with 500 keV Ar ions with *F*_*on*_ = 1.7 × 10^13^ cm^−2^ s^−1^ and *t*_*on*_ = 1 ms. Open symbols show data points for 3*C*-SiC irradiated at 100 °C with 500 keV Ar ions with *F*_*on*_ = 1.9 × 10^13^ cm^−2^ s^−1^ and *t*_*on*_ = 1 ms, taken from ref. 11.

The *τ*(*T*) dependence is shown in Fig. 4, revealing a non-monotonic behavior, when *τ* first rapidly increases with *T*, reaches a maximum at 100 °C, followed by a monotonic decrease for 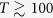 °C. Also plotted in Fig. 4 is the *T* dependence of the DA efficiency (*ξ*), which we define as before^17^: 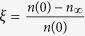. As discussed in detail recently^19^, for our choice of *F*_*on*_ and *t*_*on*_, *ξ* is the magnitude of the dose rate effect; i.e., the difference between *n* for continuous beam irradiation with dose rates of *F* = *F*_*on*_ and *F* → 0. It is seen from Fig. 4 that, in contrast to the *τ*(*T*) dependence, *ξ* increases monotonically with *T* in the *T* range of 25–175 °C. At ~175 °C, *ξ* reaches a maximum of ~80%, followed by a slight decrease at 250 °C. Hence, both *τ*(*T*) and *ξ*(*T*) dependencies of Fig. 4 are non-trivial, reflecting the complexity of defect interaction processes.

Values of *τ* revealed by Fig. 4 are in the range of ~1–5 ms. These are orders of magnitude larger than the predictions of molecular dynamics (MD) and kinetic Monte Carlo simulations discussed in our recent paper^11^. This highlights both the well-known limitations of MD to model long-time defect evolution and the currently limited understanding of radiation defect dynamics in SiC. Interestingly, Fig. 4 also shows one data point available for 3*C*-SiC at 100 °C (taken from our recent work^11^), revealing a larger *τ* for 4*H*-SiC than for 3*C*-SiC. This finding is not unexpected, given that DA processes depend on the type and properties of lattice-structure-specific point and extended defects^11^,^12^. Values of *τ* for SiC (Fig. 4) are also comparable to those for Si measured previously for room *T* bombardment with different ions (~4–13 ms)^17^,^19^. Future theoretical studies of defect interaction bench-marked against our pulsed beam data are needed to find out if such similarity in *τ* values reflects similarity in defect relaxation processes in different materials. Moreover, further systematic measurements of the *τ*(*T*) dependencies for 3*C*-SiC and 6*H*-SiC will be interesting, revealing similarities and differences in DA of the three main commonly available SiC polymorphs.

A non-monotonic *τ*(*T*)-dependence of Fig. 4 reveals the existence of a transition temperature (*T*_*r*_) of ~100 °C. Such a *T*_*r*_ of 100 °C is not evident from the *T* dependence of the damage buildup (as shown in Fig. 1 and in numerous previous studies^5^,^6^,^7^,^8^,^10^,^12^,^15^). Neither is it revealed by the *ξ*(*T*) dependence that, as mentioned above, reflects the *T* dependence of the dose rate effect, also studied previously in traditional measurements with continuous ion beams (or unintentionally scanned beams due to rastering)^13^,^15^. A special role of *T* ~ 100 °C has, however, been noted in a number of previous studies of all three main polymorphs of SiC: 4*H*-SiC^14^, 6*H*-SiC^22^,^23^,^24^,^25^, and 3*C*-SiC^26^,^27^,^28^,^29^, evidenced either as the onset of post-irradiation defect annealing^22^,^23^,^24^,^26^,^27^,^28^, as a rapid decrease in the cross-section of damage production^25^, as a qualitative change in the radiation resistance of nanocrystalline SiC^29^, or as a maximum of the concentration of open-volume defects monitored by positron annihilation spectroscopy^14^. The non-monotonic *τ*(*T*)-dependence of Fig. 4 unambiguously shows that qualitatively different defect relaxation processes are dominant above and below a transition temperature (*T*_*r*_) of ~100 °C.

**Figure 5:**
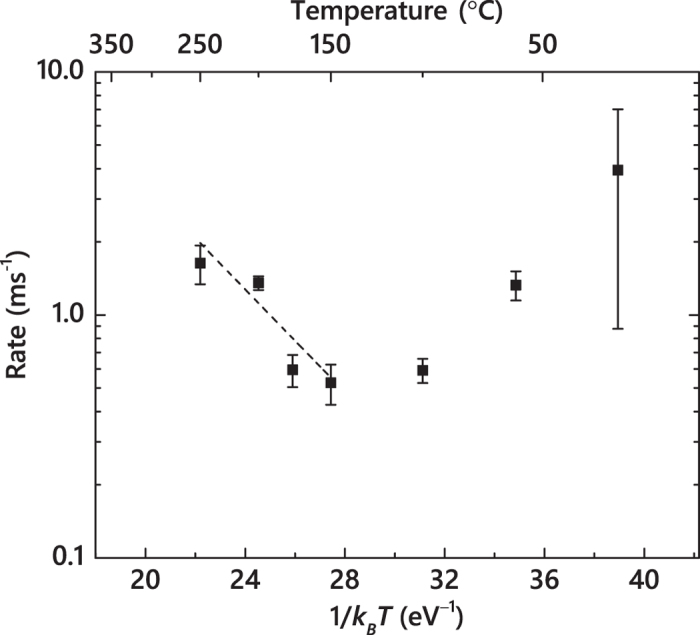
Arrhenius plot of the DA rate defined as 1/(*τ*(*n*(0) − *n*_*\infty*_)) for a second order decay process. The straight line shows results of linear fitting, revealing an activation energy of 0.25 ± 0.05 eV for the temperature range of 150–250 °C.

In order to better understand the mechanism of DA, in Fig. 5, we replot the *τ*(*T*) dependence from Fig. 4 in Arrhenius coordinates, with the DA rate defined as 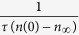, and with *k*_*B*_*T* having the usual meaning. For the second order decay process 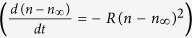, the decay time constant (*τ*) depends on the initial concentration of mobile defects, and the decay rate is 
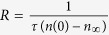
. A regime with a negative effective activation energy (*E*_*a*_) is clearly seen in Fig. 5 for *T* ≲ 100 °C. A negative *E*_*a*_ combined with an increase in *ξ* for *T* ≲ 100 °C (see Fig. 4) could suggest the dominance of thermally activated processes of defect de-trapping. Indeed, processes of defect trapping and de-trapping that do not involve defect recombination result in an increase in the effective lifetime of mobile defects and, hence, a reduced DA rate characterized by the negative *E*_*a*_.

At *T* ≳ 100 °C, thermally activated processes of defect recombination appear to dominate when *τ* decreases with increasing *T*. An Arrhenius regime with a positive *E*_*a*_ is seen for the *T* range of 150–250 °C. Linear fitting of the data gives an *E*_*a*_ and a pre-exponential factor of 0.25 ± 0.05 eV and (5.23 ± 0.62) × 10^2^ Hz, respectively. This *E*_*a*_ value is much smaller than an *E*_*a*_ of 1.3 eV obtained by Kuznetsov *et al*.^13^ by the analysis of the *T*-dependence of the dose rate effect in the *T* range of 20–225 °C based on the method proposed by Schultz and co-workers^30^, which is equivalent to the analysis of the *ξ*(*T*) dependence of Fig. 4. Such an apparent inconsistency in *E*_*a*_ could be attributed to limitations of the method of Schultz *et al*.^30^ Indeed, *ξ* (i.e., the magnitude of the dose rate effect) reflects the fraction of ballistically-generated Frenkel defects that participate in DA processes for any given *F*_*on*_ rather than the *rate* of defect interaction. Our *E*_*a*_ of ~0.25 eV is consistent with *E*_*a*_ values for 6*H*-SiC reported by Weber *et al*.^7^,^31^ from the analysis of the *T* dependence of the amorphization dose based on the empirical model of Morehead-Crowder^32^. Dedicated modeling and simulation studies are currently needed to better understand the atomistics of DA and to correlate the apparent activation energies measured here with energetic barriers of specific defect migration and interaction processes in SiC polymorphs.

In conclusion, we have used the pulsed beam method to measure the temperature dependence of both the characteristic time constant (*τ*) and the efficiency of DA in the Si sublattice of 4*H*-SiC bombarded with 500 keV Ar ions in the temperature range of 25–250 °C. Results have revealed a non-monotonic *T*-dependence of *τ* with a maximum at 100 °C, indicating a change in the dominant DA mechanism at ~100 °C. The Arrhenius regime in the *T* range of 150–250 °C is described by an activation energy of ~0.25 eV. The details of radiation defect dynamics revealed in this work have important implications for understanding, predicting, and controlling radiation damage in SiC and for benchmarking future theoretical efforts.

## Methods

High purity semi-insulating (0001) 4*H*-SiC single crystals (with a diameter of 100 mm, a resistivity of ≳10^9^ Ω cm, and a nominal micropipe density of <60 cm^−2^), obtained from Cree, Inc., were bombarded with 500 keV Ar ions at 7° off the [0001] direction in the *T* range of 25–250 °C. The crystal quality of as-received 4*H*-SiC wafers was verified by measuring a minimum 2 MeV He ion channeling yield of ~1.6%, consistent across the wafer. The 4 MV ion accelerator (National Electrostatics Corporation, model 4UH) at Lawrence Livermore National Laboratory was used for both ion irradiation and ion beam analysis. To improve thermal contact, the samples were attached to the Ni sample holder with conductive Ag paste. The sample holder temperature was monitored with a chromel/alumel thermocouple (with an accuracy of ±1 °C). All irradiations were performed in a broad beam mode^17^. Irradiated areas were ~4 × 5 mm^2^. Ion beam pulsing was achieved by applying high voltage pulses to a pair of parallel plates deflecting the beam in the vertical direction off the final beam defining aperture so that the total dose was split into a train of equal square pulses each with an *F*_*on*_ of ~(1.7 ± 0.05) × 10^13^ cm^−2^ s^−1^ and duration *t*_*on*_ = 1 ms. The adjacent pulses were separated by time *t*_*off*_, which was varied between 1 and 100 ms. For irradiation at *T* ≳ 200 °C that required larger total doses, *t*_*off*_ was limited to 20 ms, which was still much larger than the DA time constants at these *T*s. A more detailed description of the experimental arrangement can be found elsewhere^11^,^19^.

The dependence of lattice damage on *t*_*off*_ was studied *ex-situ* at room temperature by ion channeling. Depth profiles of lattice disorder in the Si sublattice were measured with 2 MeV He ions incident along the [0001] direction and backscattered into a detector at 164 ° relative to the incident beam direction. The spot size of the He beam was ~1.5 × 1.5 mm^2^. Spectra were analyzed with one of the conventional algorithms^33^ for extracting the effective number of scattering centers (referred to below as “relative disorder”). Values of averaged bulk disorder (*n*) were obtained by averaging depth profiles of relative disorder over 10 channels (~25 nm) centered on the bulk damage peak maximum. Error bars of *n* are standard deviations. Error bars of *τ*, *ξ*, and the DA rate are standard errors of the non-linear fitting described above.

The depth profile of vacancies was calculated with the TRIM code (version SRIM-2013.00, full cascade calculations)^34^ with an atomic concentration of SiC of 9.64 × 10^22^ atoms cm^−3^ (ref. 3) and threshold energies for atomic displacements of 20 and 35 eV for C and Si sublattices, respectively^35^. To convert to DPAs (at the depth corresponding to the maximum of the nuclear energy loss profile), ion doses in 10^14^ Ar ions cm^−2^ are multiplied by 0.0898. The projected range and straggle of 500 keV Ar ions are ~320 and ~70 nm, respectively.

## Additional Information

**How to cite this article**: Aji, L. B. B. *et al*. Non-monotonic temperature dependence of radiation defect dynamics in silicon carbide. *Sci. Rep*. **6**, 30931; doi: 10.1038/srep30931 (2016).
